# The Effect of Zeolite Na-X and Clinoptilolite as Functional Fillers on the Mechanical, Thermal and Barrier Properties of Thermoplastic Polyurethane

**DOI:** 10.3390/molecules30020420

**Published:** 2025-01-20

**Authors:** Nello Russo, Letizia Verdolotti, Giuseppe Cesare Lama, Federica Recupido, Barbara Liguori, Maria Oliviero

**Affiliations:** 1 Institute of Polymers, Composites and Biomaterials, National Research Council (IPCB-CNR), Portici, 80055 Naples, Italy; nellorusso@cnr.it (N.R.); giuseppecesare.lama@cnr.it (G.C.L.); federica.recupido@cnr.it (F.R.); maria.oliviero@cnr.it (M.O.); 2 Department of Chemical, Materials and Industrial Production Engineering, University of Naples Federico II, 80025 Naples, Italy

**Keywords:** thermoplastic polyurethane, zeolite Na-X, clinoptilolite, composites, functional properties

## Abstract

To obtain sustainable food packaging materials, alternatives to traditional ones must be researched. In this work, two different kinds of zeolites, i.e., a natural one, Clinoptilolite, and a synthetic one, Zeolite Na-X, were mixed with thermoplastic polyurethane for the fabrication of composites. Composite films were prepared via a hot mixing stage and then by means of a hot compression molding process. Several TPU/zeolite composites were produced with a filler concentration ranging from 5% to 10%wt. Finally, the obtained films were characterized by Fourier Transform Spectroscopy (FT-IR, ATR), thermal analysis (TGA and DSC), frequency sweep test, scanning electron microscopy (SEM), mechanical tensile test and oxygen permeability test. For both fillers and at all concentrations, the inclusion of zeolites significantly influenced the analyzed properties. In the TPU/zeolite composites, an overall enhancement was observed compared to the neat polymer, attributed to improved processability, superior barrier properties and the potential to create active materials by loading zeolite combined with various chemicals for specific applications. These findings suggest that the resulting composites hold considerable promise for applications in the food packaging sector.

## 1. Introduction

Even though commodity plastic products offer good barrier properties, making them suitable for use in the food packaging sector, the current focus is on replacing them with innovative materials to address the pressing challenge of sustainability. Traditional polymers face critical drawbacks, including limited recyclability and potential toxicity [1,2,3]. For instance, polyvinyl chloride (PVC) is extensively used as cling film in food packaging, but it is not recyclable and may pose toxicity risks due to its chemical composition. Indeed, PVC is a rigid and brittle material which can be rendered flexible, making it feasible as a cling film, via the use of specific plasticizers, like di(2-ethylhexyl) phthalate (DEHP). However, this kind of chemical can leach into food, resulting in a reduction of the product’s shelf life and in an increased risk for human health [4,5,6]. Consequently, there is a pressing need for alternative materials that are suitable for food packaging, i.e., that can ensure safer and longer preservation of food.

Thermoplastic polyurethanes (TPUs) are a large group of polymeric materials that exhibit the properties of soft elastomers and hard plastics at the same time, thanks to their chemical nature. Indeed, TPU is defined as a block copolymer which consists of soft (polyols) and hard (isocyanate) segments that form a two-phase microstructure. These two moieties are intrinsically incompatible; because of that, the hard segment is prone to aggregating, forming hard domains, whilst soft segments are responsible of the formation of amorphous regions [7]. Because of their peculiar chemical structures, TPUs are tunable by modulating the chemical nature and the disposition of the above-mentioned segments, making these kinds of materials suitable for a wide range of fabrication methods and applications [8]. To the best of our knowledge, there are few works concerning the use of TPU for food packaging. El-Nawasany et al. [9] prepared a nanocoating made of magnetic nanoparticles (Fe_3_O_4_) dispersed in a TPU nanofiber to enhance the shelf-life of dairy products, while Moustafa et al. [10] employed a polyurethane/chitin/rosin composite reinforced with ZnO-doped SiO_2_ nanoparticles to produce green packaging. Finally, Wu et al. [11] developed a spherical AgNP-PLA microsphere/PLA-thermoplastic polyurethane hierarchical antibacterial film that was tested as a packaging material for strawberries.

In composite materials, the final properties strongly depend on several factors, such as the concentration of the chosen fillers, as well as their dimension and shape (particulate, fibrillar, mat etc.), their compatibility with the polymer and, more generally, the effect that the filler can have on the matrix [12,13]. The addition of a filler can provide a simple mechanical reinforcement of the matrix and/or improve its functional properties, resulting in an active material that is suitable for a specific application. Several types of fillers are commonly used to enhance the properties of matrix materials. Among them, inorganic fillers like zinc oxide (ZnO), titanium dioxide (TiO_2_), silica (SiO_2_), diatomite, MXenes and zeolites are the most widely used in several settings because of their versatility in terms of their chemistry, their high thermal stability, their mechanical reinforcement effect [14,15], their antimicrobial properties [16,17], their ability to be employed as electrodes [18,19] and their fire safe properties [20]. Recently zeolites have received a lot of attention due to their unique and tunable characteristics [21,22,23]. Zeolites are volcanic materials which are chemically defined as silica aluminate hydrates of an alkali or alkali earth metal, whose chemical formula is:

M_x/z_ · [Si_1-x_ Al_x_ O_2_] · yH_2_O

where M is a metal, “z” is its valence and “y” is the number of water molecules. This kind of material is characterized by a three-dimensional network of silica and alumina tetrahedra that allows the formation of cavities in which the previously mentioned metals (Na, K, Mg, Ca and Fe) and water molecules can be found. Zeolites’ main feature is their wide availability in nature, coupled with the possibility of their synthetic production. Moreover, their high porosity makes them suitable for many applications, e.g., as molecular sieves, ion exchangers or water and heavy metal adsorbents [24,25,26].

Zeolites have been employed in several matrixes, like HDPE [27], ultra-high-molecular-weight polyethylene (UHMWPE) [28], ethylene vinyl alcohol (EVOH) [29], polypropylene (PP) [30] and polycarbonate (PC) [31] for various applications. Huang et al. [32] developed a LDPE/linear LDPE/zeolite H-β composite for the production of packaging material for strawberries. Boschetto et al. [33] employed the same matrix made of LDPE but, in this case, silver exchanged-zeolite Y was used. Finally, both Souza et al. [34] and do Nascimiento Sousa et al. [35] employed clinoptilolite (CLN) (the first in cassava starch and the latter in chitosan) for a food packaging application. Due to the biocompatibility of zeolites, they are attractive materials for use with the above-mentioned matrixes for biomedical and packaging purposes. As reported by the authors, following the addition of the selected filler, no detrimental effect was observed in the polymers in which it was embedded. Furthermore, many properties were enhanced, like wear resistance, which is a fundamental requirement for prosthetic devices. Lastly, the possibility of easily functionalizing these materials with active compounds, like antimicrobic and antioxidant agents (crucial for food preservation) and their good barrier properties make them very attractive for the food packaging sector.

There are very few works in the scientific literature about TPU/zeolite composites. Ghobadi et al. [36] focused on the mechanical and thermal properties of TPU/Zeolite Na-X composites, while Lei et al. [37] developed an electrospun TPU/Cu-loaded zeolite Na-X composite membrane for biomedical use. Lastly, Yildirim et al. [38] produced electrospun TPU/CLN scaffolds to be employed in tissue engineering. None of the reported TPU/zeolite composites was meant to be used in food packaging, meaning that this literature gap is open for deeper investigations.

In this work, two kinds of TPU composites were produced, i.e., using a synthetic zeolite (Na-X) and a natural one (Clinoptilolite), obtained via a two-step procedure consisting of a hot mixing stage followed by a hot compression molding step. The choice of these two kinds of zeolites was made for comparative purposes. In addition, CLN was chosen because of its high purity, i.e., it does not need any further purification process.

Several characterizations were carried out to evaluate the chemical, mechanical, thermal and functional properties of each composite to assess their suitability for food packaging applications.

## 2. Results

### 2.1. Chemical Properties

**Figure 1:**
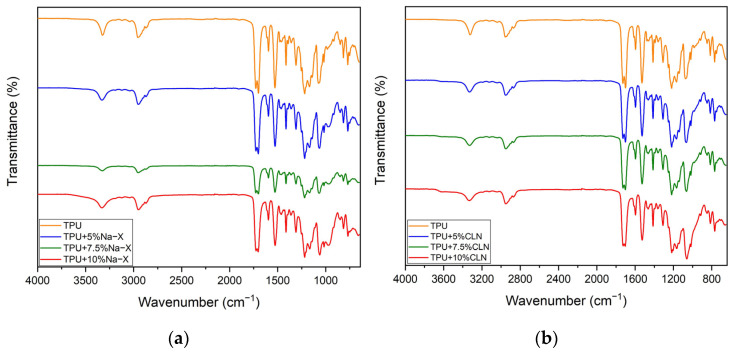
ATR spectra of (**a**) TPU + Na-X and (**b**) TPU + Clinoptilolite composites.

Figure 1a,b show the ATR spectra of TPU+Na-X and TPU+CLN composites at various concentrations, respectively. The spectra of zeolite Na-X and CLN particles [39,40,41,42,43] are reported and discussed in the Supporting Information (SI) Section (Figure S1) for the sake of brevity. A spectroscopic analysis via TPU revealed the presence of peaks at 3324, 2956 and 2871 cm^−1^ that corresponded to the symmetric stretching of -NH and -CH_2_ groups. The lack of a peak in the region from 2500 to 2000 cm^−1^, which would be related to the presence of residual isocyanate (-N=C=O), indicated that the supplied material had been totally polymerized [44,45]. Peaks at 1728 and 1700 cm^−1^ indicated the stretching of free and hydrogen bonded C=O groups, respectively. Amide II (C-N + δN-H) and amide III (C-N + C=O) combined stretching were represented by peaks at 1529 and 1254 cm^−1^, while the peaks at 1478, 1458, 1413 and 1360 cm^−1^ corresponded to the bending vibration of -CH_2_. Finally, ester (CO-O-C) and ether (C-O-C) group bending vibration peaks were located respectively at 1075 and 770 cm^−1^ [46,47]. From the ATR spectra of the TPU/zeolite composites, it can be stated that for both fillers, there was no evidence of any kind of chemical interaction between TPU and zeolites, because no new peaks were observed in the reported spectra. For the TPU/Na-X spectra, a shoulder at 960 cm^−1^ indicated asymmetric stretching of the T-O group, whilst for the TPU/CLN composite, the peak at 1019 cm^−1^ (ascribed to the second overtone of the -NH group) overlapped the absorption band of CLN, related to O-T-O symmetrical stretching.

### 2.2. Morphological Properties

**Figure 2:**
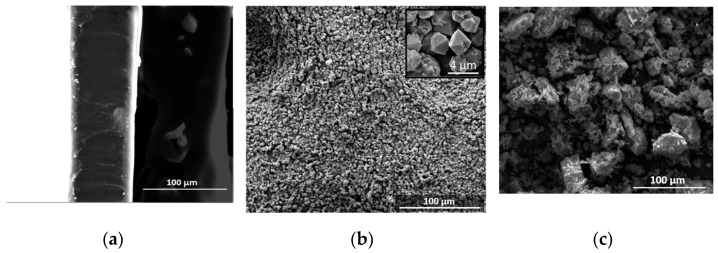

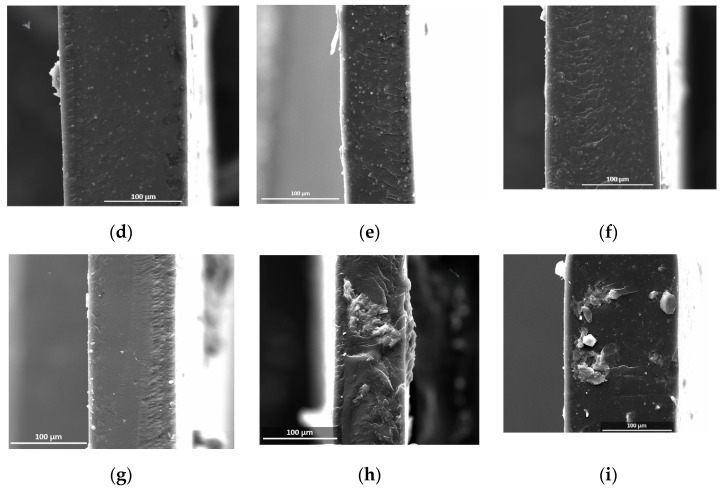
SEM images of (**a**) TPU, (**b**) Zeolite Na-X, (**c**) CLN, (**d**) TPU + 5% Na-X, (**e**) TPU + 7.5% Na-X, (**f**) TPU + 10% Na-X, (**g**) TPU + 5% CLN, (**h**) TPU + 7.5% CLN and (**i**) TPU + 10% CLN.

SEM images of zeolite Na-X, CLN and TPU, as well as of TPU+Na-X and TPU+CLN composites, are shown in Figure 2. The SEM micrograph of the TPU film shows a smooth surface morphology (Figure 2a). The Na-X zeolite had a regular octahedron-like shape and homogeneous grain size (Figure 2b), around 2.5 μm, due to its synthetic nature. On the other hand, CLN, being a milled tuff, showed a wide polydisperse grain size, along with a more irregular particle shape (Figure 2c), up to 280 μm.

Collected XRD patterns showed that both zeolitic fillers retained their crystalline structure (see Figures S2 and S3 in Supplementary Materials).

As can be noted in the composite SEM micrographs, the dispersion degree of the zeolite was not effective at all weight concentrations. Indeed, depending on the zeolite, the occurrence of a segregation phenomenon took place when a certain filler content was exceeded (above 5% for zeolite Na-X, and above 7.5% for CLN; see Figure 2d–h). This segregation can be seen as an agglomeration of the filler that reacted more favorably with itself rather than with the polymer.

### 2.3. Thermal Properties

**Figure 3:**
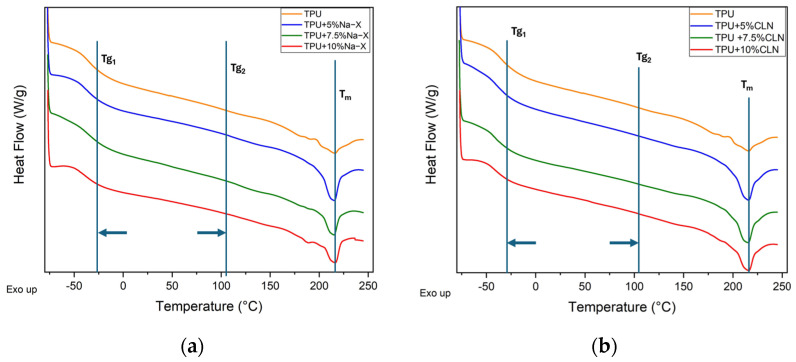
DSC curves for (**a**) TPU+Na-X and (**b**) TPU+CLN composites.

DSC second heating curves of the TPU + Na-X and TPU + CLN composites are reported in Figure 3a,b, respectively. The first heating scan was used to cancel the thermal history of the samples. Also in this case, the DSC curve of TPU was added to both sets of materials. The main thermal parameters recovered by the analysis of these curves are reported in Table 1. TPU has two glass transition temperatures (T_g1_ at −33 °C and T_g2_ at 104 °C), each referring to the glassy state originated by soft (T_g1_) and hard (T_g2_) segments, and a melting temperature (T_m_) of about 214 °C [48]. Based on Tg_1_ value, TPU behaves like a soft material at room temperature because the soft phase is in a rubbery state. For both kinds of fillers, their addition resulted in a decrease of Tg_1_ and an increase of Tg_2_, while T_m_ was not changed. This variation of Tg_1_ caused the TPU/zeolite composites to be rubbery, even at lower temperatures, with respect to neat TPU, thus making them suitable for use in colder environments without any embrittlement caused by the transition to the glassy state. An overall increase in melting enthalpy was recorded (higher at the lowest filler concentration). Therefore, the effect of zeolite within the TPU matrix consisted of the enhancement of the semi-crystalline fraction in the hard segments because of the nucleating effect of the filler resulting from a concomitant increase of Tg_2_. At the same time, the free volume of the polymers changed; this caused Tg_1_ to shift to lower values [49,50]. As can be noted, the addition of fillers resulted in variations in the glass transition temperatures and melting enthalpies; this may have been due to the presence of zeolitic structures which affected the nucleation phenomenon in a different way. Regardless of the concentration, the melting enthalpy of the composites was higher than that of neat TPU but it tended to decrease based on the filler concentration. This may have been due to the progressive agglomeration and phase segregation promoted by the filler, as already highlighted in the SEM analysis. However, despite this phenomenon, the well dispersed zeolite fraction still acted as a nucleating agent.

**Figure 4:**
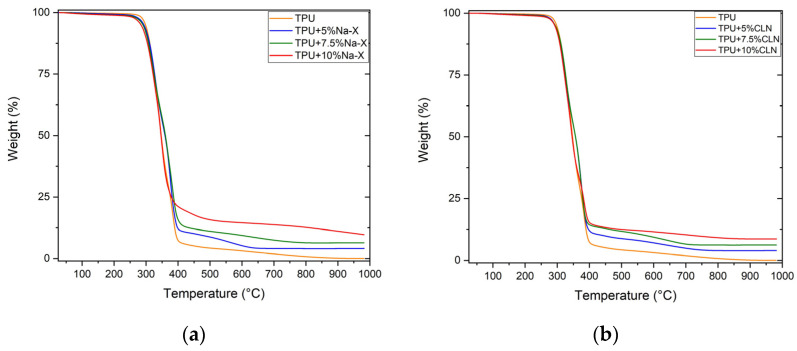
(**a**) TGA and curves of TPU + Na-X, (**b**) TGA curves of TPU + CLN composites.

TGA curves of TPU + Na-X and TPU + CLN composites are reported in Figure 4a,b, respectively, along with thermograms of pristine TPU. TGA of both pristine zeolite Na-X and CLN were acquired (Figure S4, Supplementary Section). The curves clearly show that the thermal decomposition of TPU and all TPU- based composites occurred in two successive steps: cleavage of urethanic bonds of hard segments with the formation of di-isocyanates and diols, followed by a condensation and polyol degradation of soft segments [51,52]. The temperatures corresponding to the maximum degradation rates in the two steps, also known as degradation peak temperatures and denoted as T_1_ and T_2_, are summarized in Table 2. Despite the stability at high temperature of zeolite Na-X and CLN, weight loss amounted to 24% and 12%, respectively; this was only ascribed to the evaporation of the adsorbed water. The addition of both zeolites induced a slight decrease in degradation temperatures that increased with concentration. This behavior could be attributed to the catalytic effect of zeolite, which generates free radicals, like hydroxyl ions, that degrade the matrix more rapidly [53]. Besides that, the residue at 1000 °C, based on char and zeolite-based filler, increased with respect to the pristine polymer. This could impart better flame retardant properties upon the polymer composites. As a hydrated silica aluminate, zeolite functions similarly to hydroxyl-based flame retardants and intumescent systems, expanding to form an insulating foam and thereby enhancing char formation during combustion. The presence of char significantly improves the flame retardant properties of the polymer composite. This is because char forms a protective layer on the material’s surface, which reduces the combustion rate and shields the underlying polymer from heat and oxygen. This protective layer acts as a physical barrier, limiting flame spread and the generation of flammable gases [54].

### 2.4. Mechanical Properties

**Figure 5:**
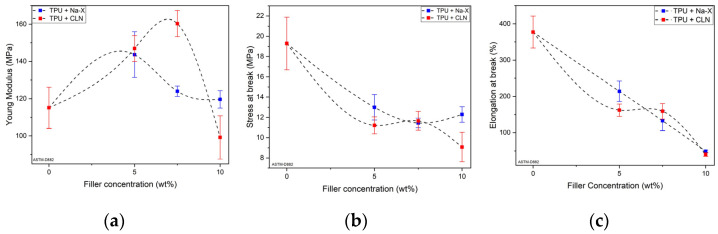
Young’s modulus (**a**), Stress at break (**b**) and Elongation at break (**c**) of the TPU + Na-X and TPU + CLN at different concentrations.

The effects of the zeolite concentration on Young’s modulus, stress and elongation at break for the TPU-based composites with Na-X and CLN are shown in Figure 5a–c, respectively (stress–strain curves are reported in Figures S5 and S6 in Supplementary Materials).

For both fillers, the Young’s modulus follows a parabolic trend in which a maximum value was reached at different Na-X and CLN weight concentrations (Figure 5a). Compared with the Young’s modulus for neat TPU, i.e.,115 MPa, Na-X composite reached a maximum value of 144 MPa at 5 wt%, with an increase of 28%, decreasing beyond this concentration. In the case of CLN, the maximum Young’s modulus value, 160 MPa, was reached at 7.5 wt% with a maximum positive variation of 39%. However, despite the subsequent decrease, this was still higher than that of TPU.

The increase of Young’s modulus at low Na-X concentrations can be attributed to the inclusion of well dispersed particles in the TPU matrix. At high Na-X concentrations, the decrease in Young’s modulus was mainly due to the saturation of the nanoparticle content. The distance between particles became shorter, inhibiting their even dispersion within the matrix and causing agglomeration. The presence of voids inside these agglomerates caused internal defects in the material and structural damage that led to a decrease in rigidity and Young’s modulus [55]. Despite the excess of Na-X, there were still some nanoparticles with good dispersion in the matrix, as highlighted in our SEM analysis. For this reason, the Young’s modulus of the composite at 10 wt% of Na-X was still slightly higher than that of TPU. Instead, as shown in the Figure 5b,c, the stress and elongation at break presented lower values across the Na-X concentration range (with respect to those of the TPU), which could be attributed to the interruption of the co-continuity of the polyeric matrix (elongation at break as a quasi-linear decrease). Up to 5 wt% of Na-X, the reduction of these mechanical parameters was ascribed to the poor interaction between the filler and TPU, which was responsible for the formation in the matrix of a filler grid without a contacted interface. At higher Na-X content, the excess of nanoparticles restricted the mobility of the molecular chains of TPU. At the same time, it induced the formation of agglomerates and voids in correspondence with the stress concentration, and damage and fractures occurred under force [56].

Similar trends for stress and elongation at break were observed for the TPU-based composites with CLN. However, in these cases, saturation was reached at higher CLN concentrations (7.5 wt%) compared to Na-X (5 wt%).

### 2.5. Barrier Properties

The oxygen barrier properties (diffusion coefficient, D, solubility coefficient, S, diffusion flux, J and permeability, P) were also assessed in the selected samples (zeolite Na-X based composites); the results are reported in Table 3. Tests performed on TPU + CLN composites did not provide data, because the oxygen permeability of the samples exceeded the instrument limit (2.3 × 10^−5^ cm^3^·m/m^2^·s·atm). Generally, the pore structure of zeolite is such that small molecules, like H_2_O, can diffuse more easily, whilst O_2_ can only migrate with difficulty [57]. As noted, oxygen permeability P of sample TPU + 7.5%Na-X was higher than that of TPU + 5%Na-X and TPU + 10%Na-X; this was due to the formation of filler aggregates, as highlighted in our SEM analysis, as reflected in a higher free volume, and hence, a higher permeability for oxygen molecules. As shown in Table 3, the addition of zeolite Na-X induced an enhancement of the oxygen barrier property with respect to pristine TPU because of the formation of a more tortuous path for the oxygen molecules, resulting in a more difficult migration of O_2_ along the matrix. Based on the variation of the oxygen permeability values and on the requirements for food packaging, as reported in Goswami et al. [58], this kind of composite film is attractive for applications in modified atmosphere packaging for fresh meat.

### 2.6. Rheological Properties

To validate the feasibility of utilizing these composite polymers as stretch films in domains such as food packaging, it is imperative to ascertain their rheological properties, given their processability through conventional technologies employed in these sectors, including extrusion, melt blending and molding.

**Figure 6:**
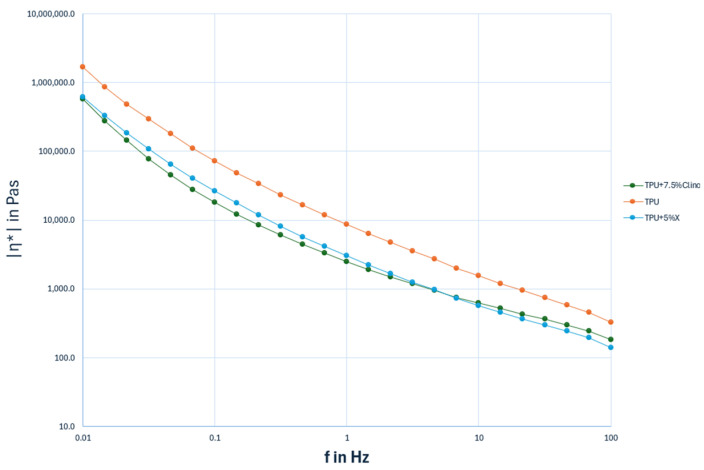
Complex viscosity |η*| vs. frequency at room temperature of TPU, TPU + 5% Na-X and TPU + 7.5% CLN. The trend lines have been added for each viscosity curve.

Generally, the processing performance of polymer melts is related to their shear flow properties. Measurements of complex viscosity |η*| were therefore performed to establish the effects of Na-X and CLN on the processability of TPU; selected results are illustrated in Figure 6. The change in complex viscosity |η*| with increasing oscillation frequency (Figure 6) illustrated how the viscosity curve was affected by the presence of two types of zeolites. All samples demonstrated a pronounced shear-thinning behavior, typical of pseudoplastic fluids, which exhibit low viscosity at high frequency and vice versa. However, all the composites showed a reduction in complex viscosity at all frequencies. This effect may be attributed to the insufficient interactions between TPU and zeolites, as well as the lack of a secondary network structure formed through the entanglement between nanoparticles and the polymer matrix [53]. In addition, the reduction of complex viscosity after the addition of the filler in both cases could be ascribed to the catalytic effect of zeolites toward the matrix, as already highlighted in our TGA analysis, during the hot mixing process, which resulted in a reduction in the length of the polymeric chains, thus making the material flow more easily.

These findings suggest that the analyzed composites could be processed using the conventional thermoplastic polymer technologies which are widely employed in the food packaging industry.

## 3. Materials and Methods

### 3.1. Materials

Thermoplastic polyurethane pellets (Ravathane^®^ 141 A93 10), with a density of 1.95 g/cm^3^, were purchased from Ravago Petrokimya (İzmir, Turkey). Na-X is a commercial FAU zeolite purchased from Alfa Aesar. Clinoptilolite is a powdered clinoptilolite-rich tuff with the commercial name IZ-CLINO, purchased from Italiana Zeoliti, Emilia-Romagna, Italy.

### 3.2. TPU-Based Composites Preparation

TPU/zeolite composite films were prepared through a two-step procedure. The first step consisted of preparing composite materials using a twin counter rotating internal mixer connected to a control unit (Rheomix 600 and Rheocord 9000, respectively, Haake, Vreden, Germany), at 220 °C, 50 rpm for 10 min. TPU pellets were first melted at 220 °C, 20 rpm for 2 min and, subsequently, zeolite was added to the mixing chamber and mixed at 50 rpm for 8 min. For each kind of zeolite, different TPU/zeolite compositions were prepared. Neat TPU was subjected to the same mixing procedure for comparison purposes. The mixing compositions and classifications of the samples are reported in Table 4.

In the second step, the materials extracted from the mixer were pressed at 225 °C and 30 MPa into films with a thickness of 0.5 mm using a hot press (P300P, Collin, Maitenbeth, Germany).

### 3.3. TPU-Based Composite Characterization

A Fourier transform infrared spectrometer (FTIR Nicolet, Thermo Scientific, Parma, Italy) was used to evaluate the chemical structure of TPU-based composites. The samples were analyzed at ambient temperature in ATR mode from 4000 to 650 cm^−1^ with a wavenumber resolution of 4 cm^−1^ and an average of 32 scans.

Morphological studies of the fractured surfaces of the composite films were carried out using an FEI Quanta 200 Field-Emission Gun (FEG) Scanning Electron Microscope (SEM) (FEI, Hillsboro, OR, USA) under vacuum with an accelerating voltage of 10–30 kV. Samples were put on an aluminum stab before undergoing a metallization process with an Au/Pd alloy via a sputtering operation (Emitech K575X) to make them suitable for this type of analysis.

In order to control the crystalline structures of the zeolitic fillers after the production process, X-ray diffraction analysis (XRD) was performed either on zeolites (Na-X and CLN or on all the TPU/zeolite composite films using a Panalytical X’Pert Pro diffractometer equipped with a PixCel 1D detector (operative conditions: CuKα1/Kα2 radiation, 40 kV, 40 mA, 2θ range from 5 to 80°, step size 0.0131 °2θ, counting time 40 s per step). Crystalline phases were identified with PANalytical HighScore software, provided with the ICDD PDF-4+ database.

The thermal behavior was studied using differential scanning calorimetry (DSC) and thermo-gravimetric analysis (TGA). Using DSC, the melting (T_m_) and glass transition (T_g_) temperatures were assessed. Approximately 8 mg of the sample was placed in a standard aluminum pan and analyzed on a DSC Q2000 (TA Instruments, New Castle, DE, USA) under inert atmosphere using a cycle of heating–cooling–heating from −80 °C to 250 °C, at a constant temperature gradient of 10 °C/min. The thermal degradation temperatures, along with the char residues, were assessed using TGA; experiments were carried out on a TGAQ500 (TA Instruments, New Castle, DE, USA) over a temperature range of 30 °C to 1000 °C at 10 °C/min under inert atmosphere.

Mechanical performance was assessed via tensile tests, performed at room temperature, according to ASTM standard D882, using a CMT 4304 Sans Testing Machine (SANS, Shenzhen, China) equipped with a 2.5 kN load cell. Force and displacement were measured by the apparatus and recorded to evaluate the Young’s modulus, as well as stress and elongation at break. Five samples for each composition were tested, and the average values were reported.

Rheological experiments were performed using a stress controlled rotational rheometer (RheoScope MARS II, Haake, Vreden, Germany) equipped with 20 mm parallel plates. The tests were conducted from 200 °C to 220 °C under nitrogen atmosphere, using a gap thickness of 0.2 mm. Frequency sweep tests from 0.01 to 100 Hz with a fixed strain of ζ = 0.1% were carried out to operate in the linear viscoelastic region.

The oxygen permeability (P), as well as diffusion coefficient (D) and solubility coefficient (S) were measured and calculated using a standard permeabilimeter (Extrasolution, MULTIPERM), at 38 °C and 50% relative humidity. Each test was carried out in duplicate.

To evaluate P, Fick’s law was used by means of Equation (1) [59]

(1) $$J=-D\frac{\partial C}{\partial x}$$

where *J* is the diffusion flux (in cm^3^/m^2^⋅s), *D* is the diffusion coefficient (in m^2^/s) and *∂C*/*∂x* is the concentration gradient. Starting from Equation (1), and considering discrete intervals for *∂C* and *∂x*, such as Δ*C* = *C*_2_ − *C*_1_ (with 1 where the concentration is higher, and 2 where the concentration is lower) and *L* as the thickness of the selected sample, respectively, Equation (1) is as follows:

(2) $$J=-D\frac{C_{2}-C_{1}}{L}$$

Moreover, considering that for gases, concentration *C* can be expressed in terms of pressure *p* using Henry’s Law (*C* = *S*⋅*p*) [60], with *S* as the solubility coefficient and *p* as the gas pressure (atm), Equation (2) is:

$$J=-DS\frac{p_{2}-p_{1}}{L}=DS\frac{p_{1}-p_{2}}{L}=P\frac{p_{1}-p_{2}}{L}$$

where *P* is defined as the relationship between diffusion and solubility in permeable materials (*P* = *D*·*S*) [61]. To evaluate *J*, *p*_2_ is considered to be equal to 0 atm, while *p*_1_ is set at 1 atm.

## 4. Conclusions

In this study, zeolite-based composites with a TPU matrix were formulated and various properties were characterized, including mechanical, thermal, morphological, chemo-structural and barrier properties. Two types of zeolites were selected: a synthetic zeolite (Zeolite Na-X) and a natural zeolite (Clinoptilolite). These were incorporated into the TPU matrix at different weight concentrations: 5 wt%, 7.5 wt% and 10 wt%.

The results indicated a significant increase in Young’s modulus, with improvements of 28% for composites containing 5 wt% Zeolite Na-X and 39% for those with 7.5 wt% CLN. However, at higher concentrations, a segregation phenomenon led to a decrease in mechanical properties.

The addition of zeolites also enhanced the oxygen barrier properties. Data showed an improvement in oxygen barrier properties compared to pristine TPU, attributed to an increase in crystallinity, as observed through DSC and FTIR analyses.

Thermogravimetric analysis (TGA) revealed that the addition of both types of zeolites resulted in a higher residue content at 1000 °C, due to the zeolite and increased char formation. This enhanced char formation, along with the zeolite, contributed to the improved flame retardant properties of the polymer composites compared to the pristine polymer.

Furthermore, rheological characterization demonstrated that TPU composites with 5 wt% Zeolite Na-X and 7.5 wt% CLN exhibited shear thinning behavior, characteristic of pseudoplastic fluids. These fluids showed low viscosity at high shear rates and high viscosity at low shear rates, facilitating the processing of materials using the thermoplastic polymer technologies commonly employed in the food packaging industry. This makes these composites suitable candidates for the development of new, highly processable packaging materials.

## Figures and Tables

**Table 1:** Main thermal parameters from DSC curves for TPU, TPU + Na-X and TPU + CLN composites.

Sample	Tg_1_ [°C]	Tg_2_ [°C]	T_m_ [°C]	ΔH_m_ [J/g]
TPU	**−33**	**104**	**214**	**2.38**
TPU + 5% Na-X	**−37**	**108**	**212**	**5.87**
TPU + 7.5% Na-X	**−38**	**116**	**214**	**4.41**
TPU + 10% Na-X	**−39**	**137**	**216**	**3.85**
TPU + 5% CLN	**−35**	**136**	**212**	**6.33**
TPU + 7.5% CLN	**−40**	**128**	**211**	**5.89**
TPU + 10% CLN	**−41**	**132**	**213**	**4.95**

**Table 2:** Degradation peak temperatures for TPU, TPU + Na-X and TPU + CLN composites.

Sample	T_1_ [°C]	T_2_ [°C]	Residue at 1000 °C/%
TPU	343	384	0.06
TPU + 5% Na-X	328	375	4.12
TPU + 7.5% Na-X	324	383	6.41
TPU + 10% Na-X	323	347	9.70
TPU + 5% CLN	324	375	3.97
TPU + 7.5% CLN	327	373	6.26
TPU + 10% CLN	326	343	8.67

**Table 3:** Oxygen diffusion coefficient (D), solubility coefficient (S), diffusion flux (J) and permeability (P) of the selected samples.

Sample	D [10^−6^ cm^2^/s]	S cm^3^(STP)/cm^3^⋅atm	J [10^3^ cm^3^/m^2^ s]	P [10^−6^ cm^3^⋅m/m^2^⋅s⋅atm]
TPU	2.53	45.8	7.70	1.16
TPU + 5% Na-X	1.97	44.7	5.94	0.88
TPU + 7.5% Na-X	2.31	45.4	8.40	1.05
TPU + 10% Na-X	2.19	42.9	6.84	0.94

**Table 4:** Mixing compositions and classifications of TPU-based composites.

Sample	TPU (%)	Zeolite Na-X (%)	Clinoptilolite (%)
TPU	100	-	-
TPU + 5% Na-X	95	5	-
TPU + 7.5% Na-X	92.5	7.5	-
TPU + 10% Na-X	90	10	-
TPU + 5% CLN	95	-	5
TPU + 7.5% CLN	92.5	-	7.5
TPU + 10% CLN	90	-	10

## Data Availability

Dataset available on request from the authors.

## Supplementary Materials

The following supporting information can be downloaded at: https://www.mdpi.com/article/10.3390/molecules30020420/s1, Figure S1: ATR spectra of Zeolite Na-X and CLN powders. Figure S2: XRD spectra of TPU + Na-X composites. Figure S3: XRD spectra of TPU + CLN composites. Figure S4: TGA curves of Zeolite Na-X and CLN powders. Figure S5: Stress-Strain curves of TPU + Na-X composites. Figure S6: Stress-Strain curves of TPU+CLN composites.
