# Supplementary material for: The Effect of Zeolite Na-X and Clinoptilolite as Functional Fillers on the Mechanical, Thermal and Barrier Properties of Thermoplastic Polyurethane
Source: Molecules. 2025 Jan 20;30(2):420. doi: 10.3390/molecules30020420 (PMC11767372; doi:10.3390/molecules30020420)
Supplement: Supplementary file 1 [file molecules-30-00420-s001.zip › molecules-3372257-supplementary.pdf]

molecules

MDPI

Article

SUPPLEMENTARY MATERIAL

The effect of zeolite Na-X and Clinoptilolite as functional fillers on the mechanical, thermal and barrier properties of Thermoplastic Polyurethane

Nello Russo [1,2], Letizia Verdolotti [1*], Giuseppe Cesare Lama [1], Federica Recupido [1] , Barbara Liguori [2*] and Maria Oliviero[1]

[1] Institute of Polymers, Composites and Biomaterials, National Research Council (IPCB-CNR) 80055 Portici, Naples, Italy; nellorusso@cnr.it (N.R); giuseppecesare.lama@cnr.it (G.C.L.); federica.recupido@cnr.it (F.R.); maria.oliviero@cnr.it (M.O.)

[2] University of Naples Federico II, Department of Chemical, Materials and industrial production engineering 80025 Naples, Italy

* Correspondence: letizia.verdolotti@cnr.it (L.V.), barbara.liguori@unina.it (B.L.)

Supplementary Materials

**SECTION 1**	FTIR CHARACTERIZATION
**SECTION 2**	XRD CHARACTERIZATION
**SECTION 3**	TGA CHARACTERIZATION
**SECTION 4**	MECHANICAL CHARACTERIZATION

SECTION 1: FTIR CHARACTERIZATION

FTIR results demonstrate (Figure S1) that both types of zeolites are significantly hydrated which is illustrated by a discrete water transmittance band in the 3500 and 1640 cm–1 region. These bands, which are centered at 3379 (OH group) and 1644 cm–1 for Na-X and 3403 and 1627 cm–1 for Clinoptilolite, refer to water molecules associated with the ions in the channels and cages in the of the zeolite structure [39] (reference of the main manuscript). The zeolite Na-X exhibits other bands appear near at 959 cm-1, 752 cm-1 and 673 cm-1 ascribed to asymmetric stretching of T-O group (where T= Si or Al), T-O-T group stretching and asymmetric stretching of T-O. For Clinoptilolite, the peaks at 1007 cm-1 and 794 cm-1 correspond to the symmetric and asymmetric stretching of O-T-O group. The other peaks, located at 722 and 671 cm-1 are characteristic of the symmetric stretching of free tetrahedral group TO_4 [40-43] (references of the main manuscript).

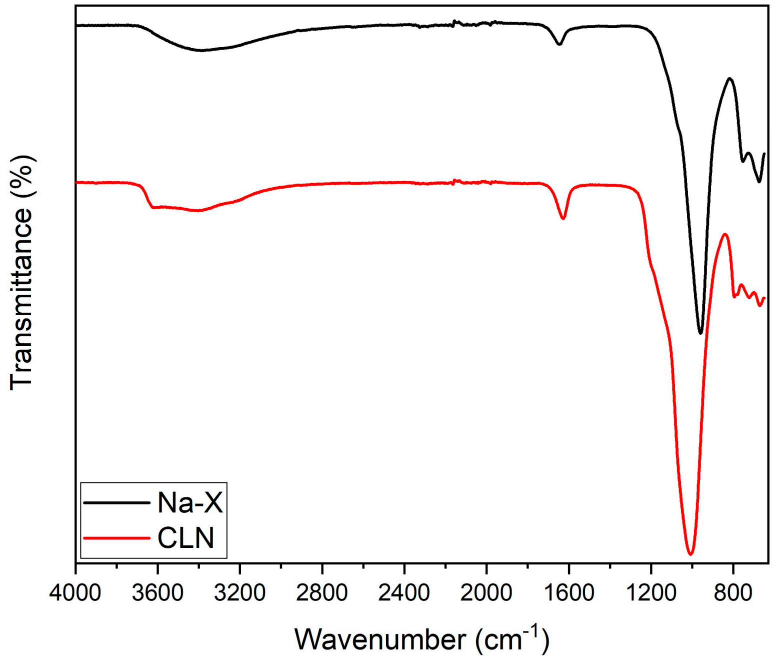

Figure S1. ATR spectra of Zeolite Na-X and Clinoptilolite powders

SECTION 2: XRD CHARACTERIZATION

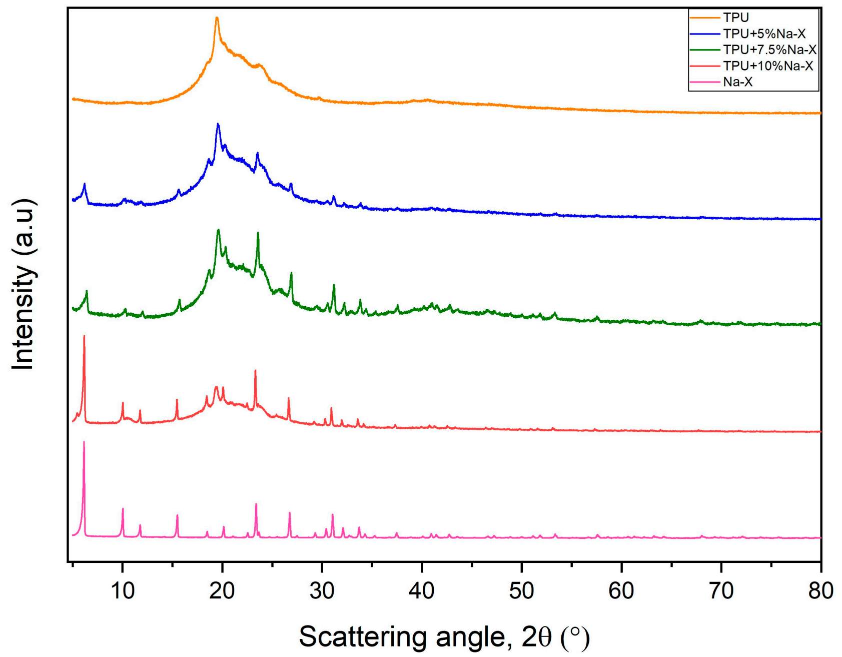

Figure S2. XRD spectra of Thermoplastic Polyurethane-TPU +Na-X composites.

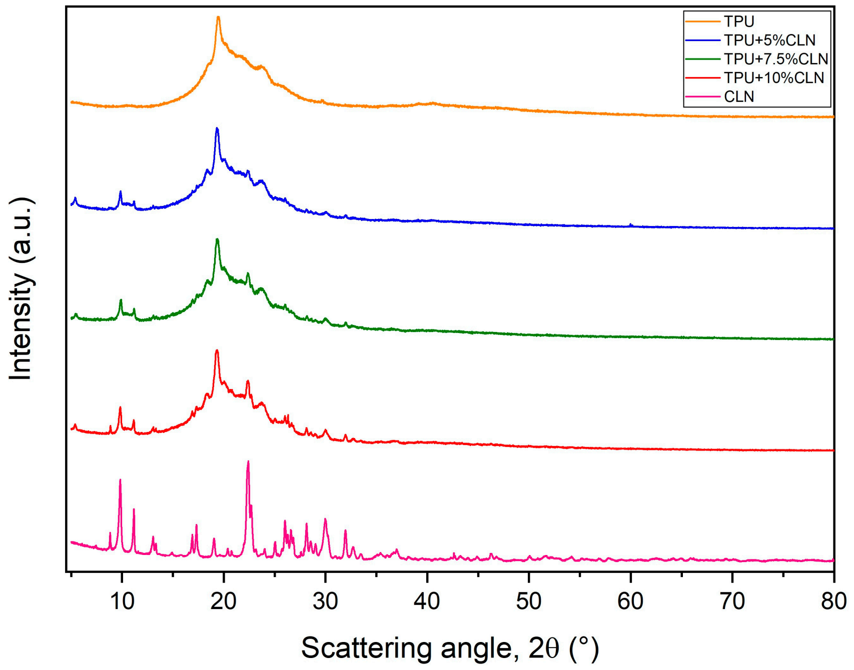

Figure S3. XRD spectra of Thermoplastic Polyurethane-TPU +CLN composites.

SECTION 3: TGA CHARACTERIZATION

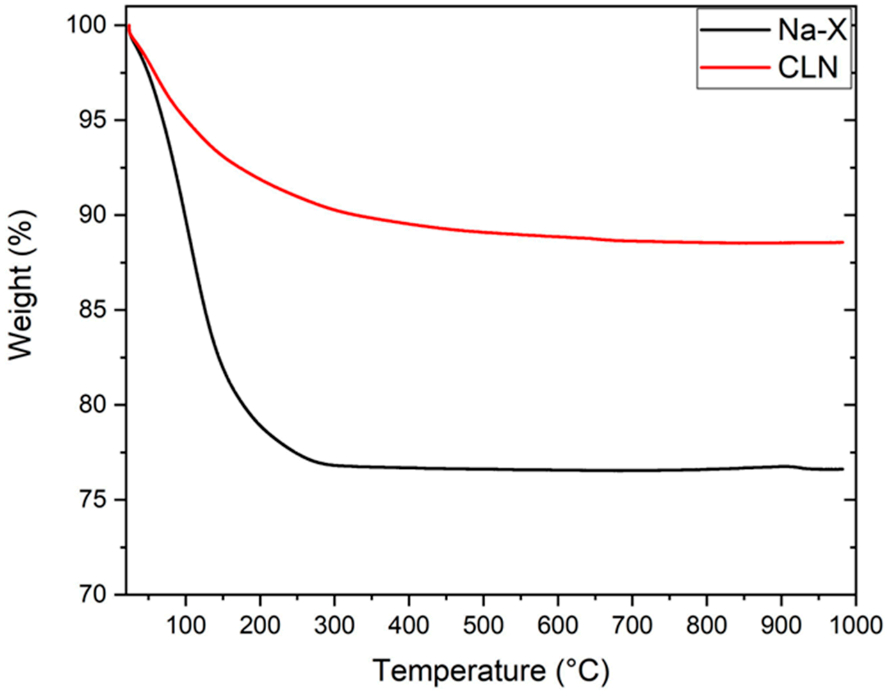

Figure S4. TGA curves of Zeolite Na-X and Clinoptilolite powders.

SECTION 4: MECHANICAL CHARACTERIZATION

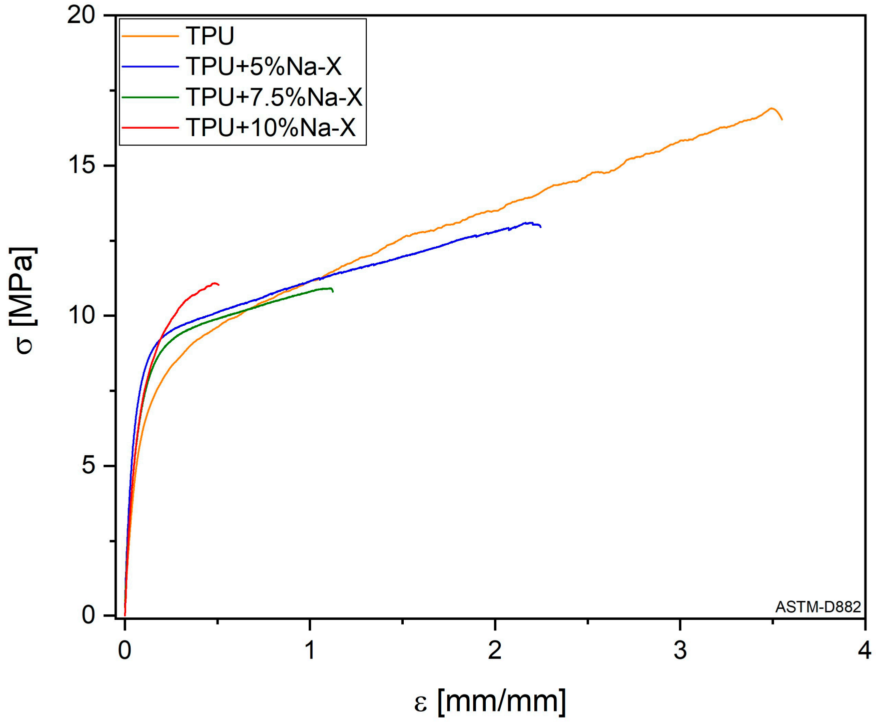

Figure S5. Stress-Strain curves of Thermoplastic Polyurethane-TPU +Na-X composites.

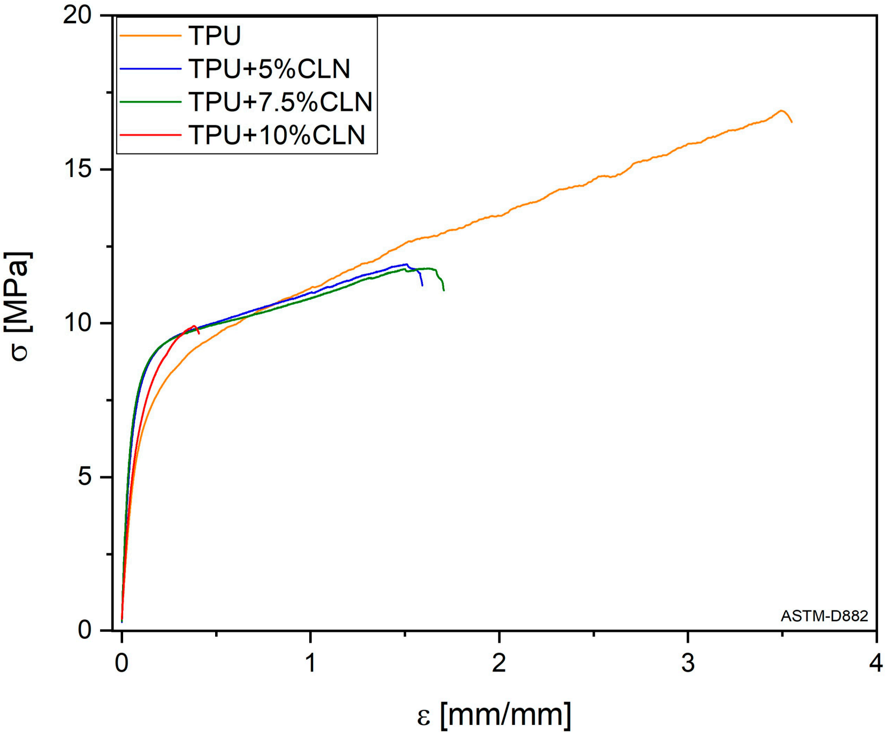

Figure S6. Stress-Strain curves of Thermoplastic Polyurethane-TPU +CLN composites.
